# Supplementary material for: A Sexually Dimorphic Role for STAT3 in Sonic Hedgehog Medulloblastoma
Source: Cancers (Basel). 2019 Nov 1;11(11):1702. doi: 10.3390/cancers11111702 (PMC6895805; doi:10.3390/cancers11111702)
Supplement: Supplementary file 1 [file cancers-11-01702-s001.pdf]

cancers

Supplementary Figures

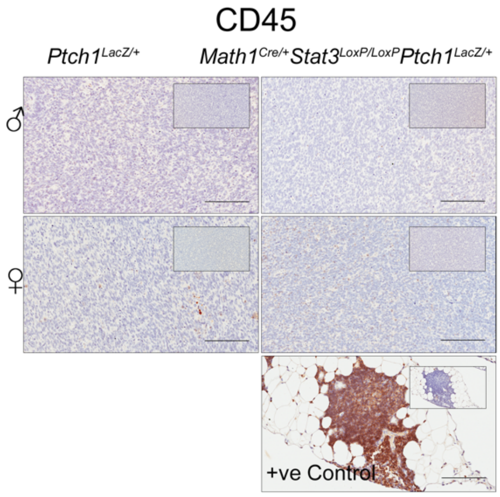

Figure S1: **STAT3 expression is not due to infiltrating immune cells in *Stat3*-deficient mice.** Immunohistochemical staining against CD45 on tumors from male and female *Ptch1*$^{LacZ/+}$control and *Math1*$^{Cre/+}$*Stat3*$^{LoxP/LoxP}$*Ptch1*$^{LacZ/+}$ mice did not reveal substantial staining in either sex or genotype. A section from mouse lung adenocarcinoma with prominent leukocytic infiltrate was stained as a positive control. Representative fields from three biological replicates (except *Math1*$^{Cre/+}$*Stat3*$^{LoxP/LoxP}$*Ptch1*$^{LacZ/+}$ males where only duplicates were available) shown. Insets show secondary antibody alone controls. Scale bar = 100 μM.

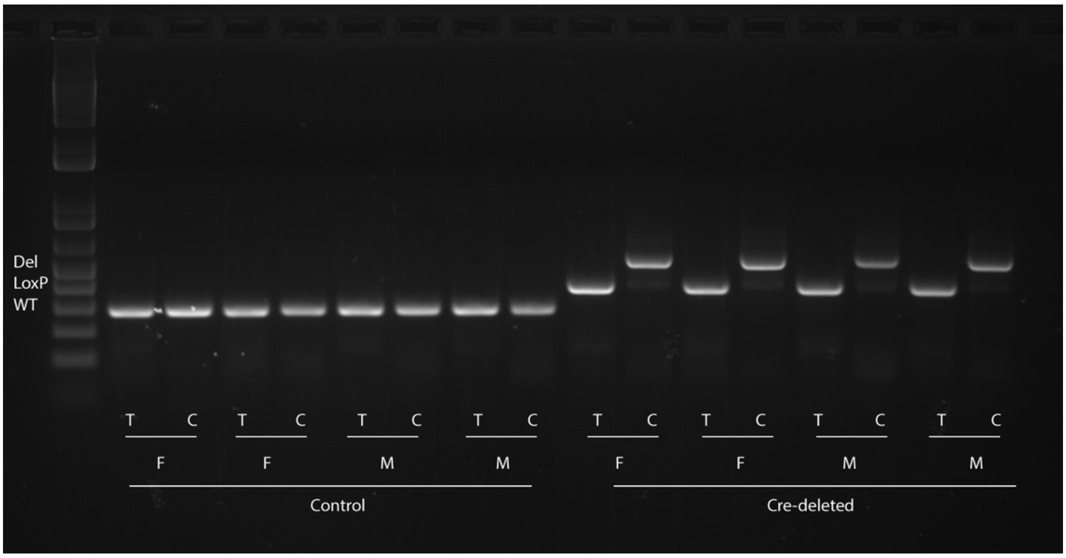

Figure S2: Cre-deletion effectively removes *Stat3* in tumor cells in males and females. Genotyping PCR of tail (T) and cerebellar tumor cells (C) was used to determine whether Cre-deletion was effective. Control female (F) and (M) mice lacking the LoxP-flanked *Stat3* allele showed the expected 250bp wild-type allele in tail and tumor. Both male and mice containing *Math1-Cre* and *Stat3-LoxP* alleles showed the expected 350bp LoxP-flanked *Stat3* allele in tail samples and the 550bp deleted *Stat3* allele in cerebellar tumor cell samples.

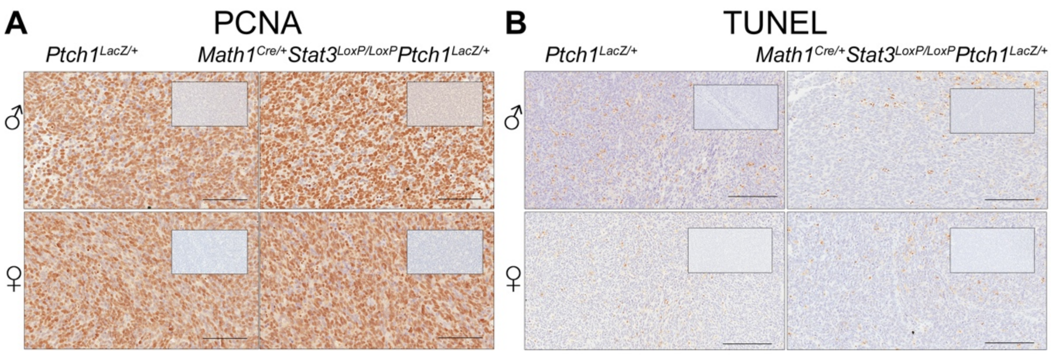

Figure S3. ***Stat3*** **Deletion does not alter proliferation or apoptosis in tumor cells**. (**A**) Male and female mice with (*Ptch1*$^{LacZ/+}$) or lacking (*Math1*$^{Cre/+}$*Stat3*$^{LoxP/LoxP}$*Ptch1*$^{LacZ/+}$) *Stat3* showed comparable proliferation as measured by expression of Proliferating Cell Nuclear Antigen (PCNA). (**B**) Mice as in A also showed comparable apoptosis as measured by TUNEL. Representative fields from three biological replicates (except *Math1*$^{Cre/+}$*Stat3*$^{LoxP/LoxP}$*Ptch1*$^{LacZ/+}$ males where only duplicates were available) shown. Insets show secondary antibody alone controls. Scale bar = 100 μM.

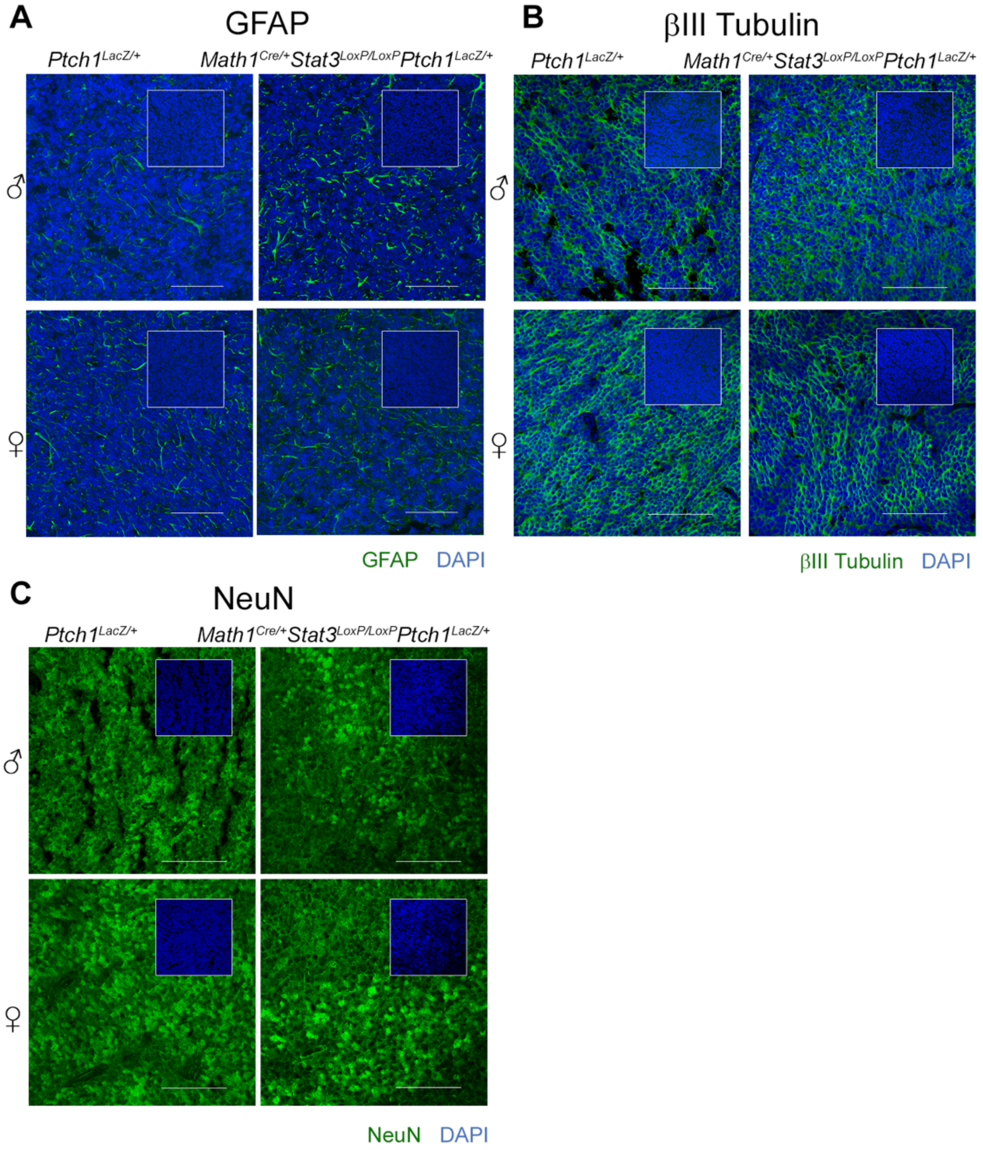

Figure S4 ***Stat3*** **deletion increases glial but not neuronal marker expression in tumor cells.** Immunofluorescent staining for (**A**) the glial marker GFAP, (**B**) the developing neuronal marker β-III Tubulin or (**C**) the terminally differentiated neuronal marker NeuN from control mice with (*Ptch1*$^{LacZ/+}$) or without (*Math1*$^{Cre/+}$*Stat3*$^{LoxP/LoxP}$*Ptch1*$^{LacZ/+}$) *Stat3* showed a small increase in GFAP staining in *Stat3*-deficient mice of both sexes. No other difference in staining patterns between genotypes or sexes were observed. Representative fields from three biological replicates (except *Math1*$^{Cre/+}$*Stat3*$^{LoxP/LoxP}$*Ptch1*$^{LacZ/+}$ males where only duplicates were available) shown. Scale bar = 100 μM.
